# Supplementary material for: Morphology and Microwave-Absorbing Performances of Rubber Blends with Multi-Walled Carbon Nanotubes and Molybdenum Disulfide
Source: Nanomaterials (Basel). 2023 May 15;13(10):1644. doi: 10.3390/nano13101644 (PMC10223461; doi:10.3390/nano13101644)
Supplement: Supplementary file 1 [file nanomaterials-13-01644-s001.zip › nanomaterials-2380862-supplementary.pdf]

Supplementary Information

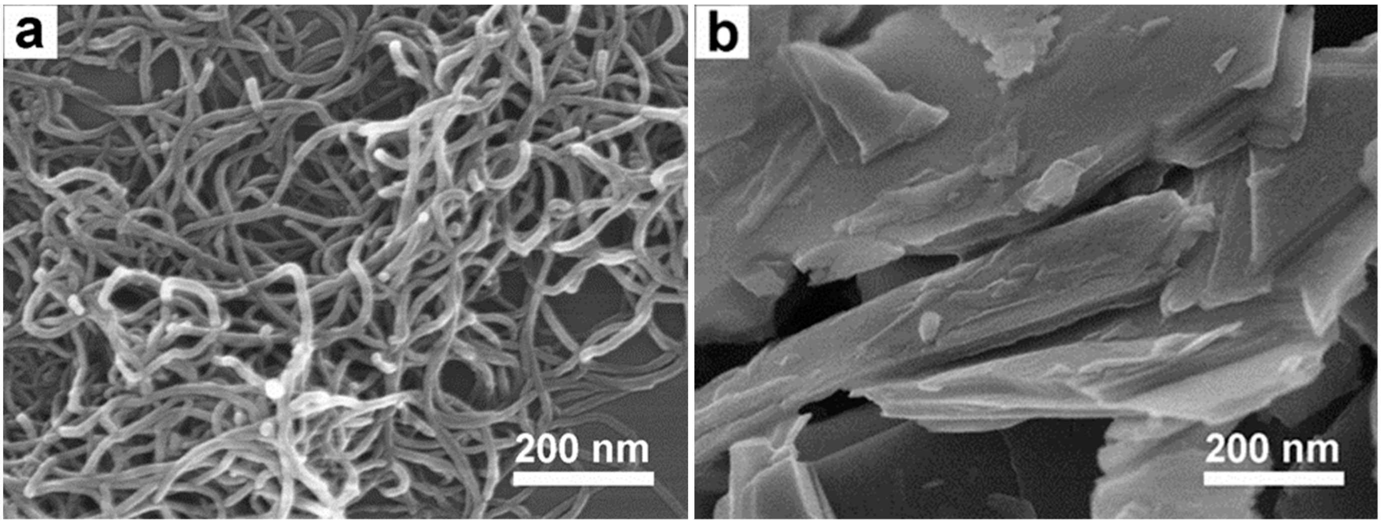

Figure S1. SEM images of (a) MWCNTs and (b) MoS_2.

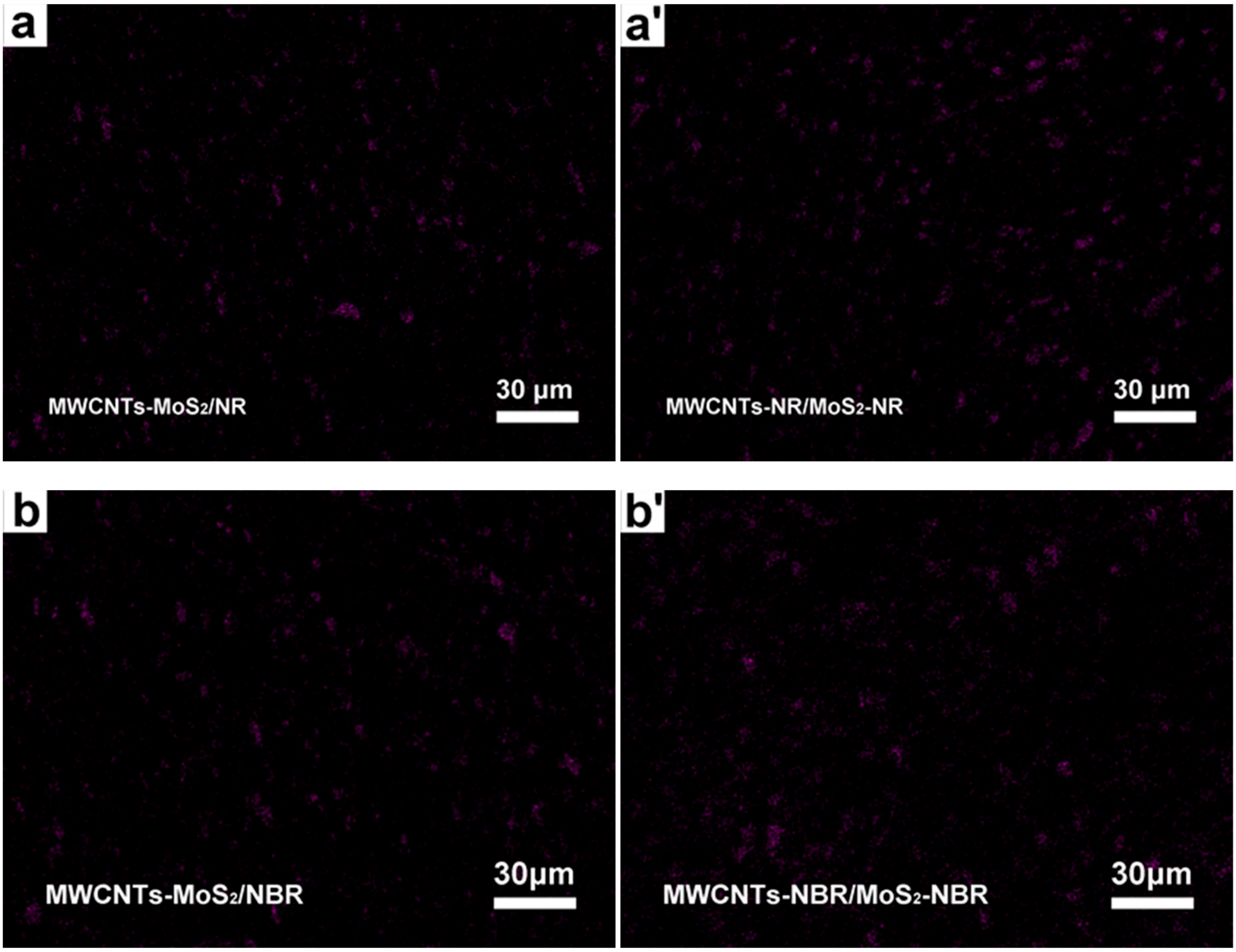

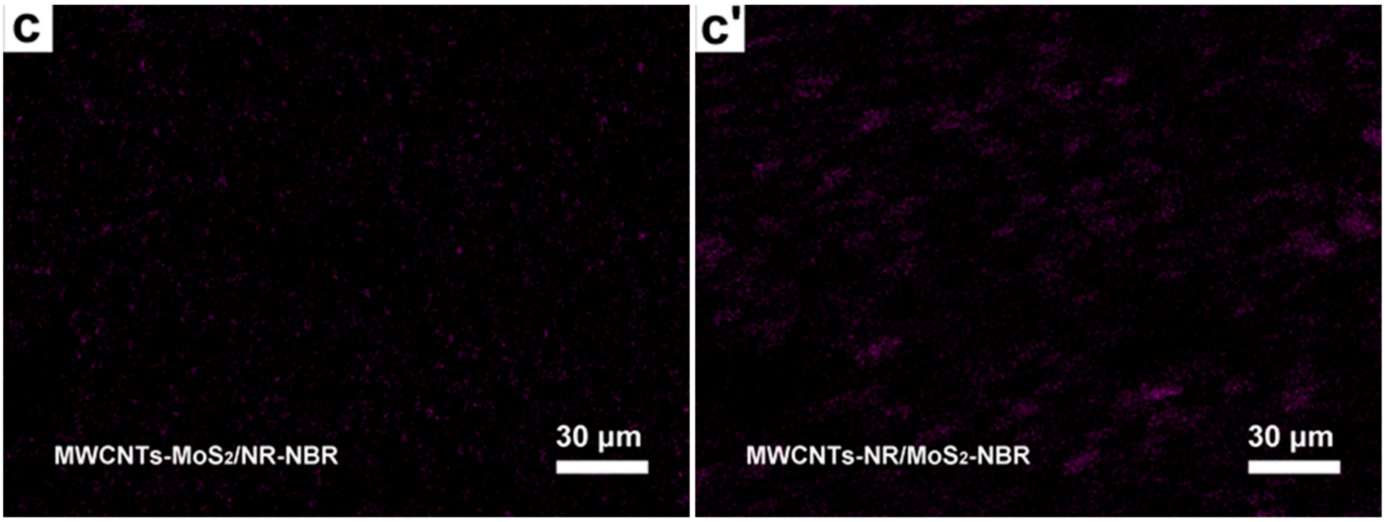

Figure S2. Element mapping of Mo for resulted composites.

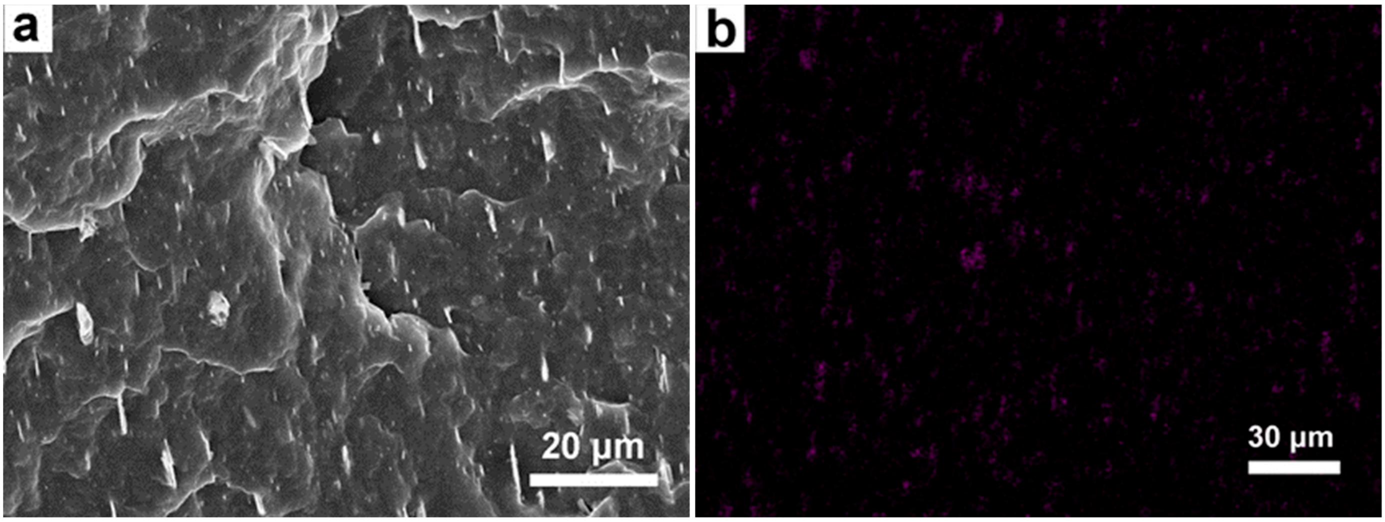

Figure S3. (a) SEM image and (b) Element mapping of Mo for MWCNTs-NBR/MoS_2-NR composite.

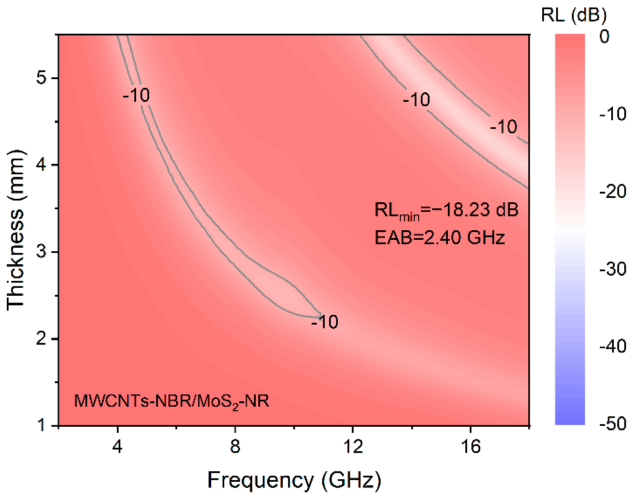

Figure S4. Frequency/thickness-depended reflection loss of MWCNTs-NBR/MoS_2-NR composite.
